# Comparative Evaluation of Bond Strength and Microleakage of Standard and Expired Composite at Resin-Dentin Interface: An *in vitro* Study

**DOI:** 10.5005/jp-journals-10005-1396

**Published:** 2017-02-27

**Authors:** Nidhi Talreja, Shilpy Singla, ND Shashikiran

**Affiliations:** 1 Postgraduate Student, Department of Pedodontics and Preventive Dentistry, People’s College of Dental Sciences and Research Centre, People’s University, Bhopal, Madhya Pradesh, India; 2 Reader, Department of Pedodontics and Preventive Dentistry, People’s College of Dental Sciences and Research Centre, People’s University, Bhopal, Madhya Pradesh, India; 3 Professor and Head, Department of Pedodontics and Preventive Dentistry, People’s College of Dental Sciences and Research Centre, People’s University, Bhopal, Madhya Pradesh, India

**Keywords:** Bond strength, Expired composites, Microleakage.

## Abstract

**Background:**

Evaluation of bond strength and microleakage caused by polymerization shrinkage provides a screening mechanism and an indication of the potential for the clinical success of composite restorations.

**Aim:**

The aim of this study was to determine the effect on shear bond strength and microleakage of standard and expired composites.

**Materials and methods:**

Selected specimens were etched using 37% phosphoric acid for 15 seconds and were randomly divided into four groups. Group I: Standard composite and bonding agent; group II: Expired composite and bonding agents; group III: Standard composite and expired bonding agent; and group IV: Expired composite and standard bonding agent. Specimens were further subdivided into two subgroups. In subgroup A, specimens were sheared with a universal testing machine, and the results were calculated in MPa; in subgroup B, specimens was sectioned longitudinally and analyzed for leakage (dye penetration) using a stereomicroscope.

**Results:**

The results of the present study showed that acceptable values for bond strength and microleakage were obtained even if one of the components of the dental resin composite is expired.

**Conclusion:**

In Indian scenario, the expired composite material may provide some assistance in compromised clinical situations. It can be used as an interim restoration and compensate for the high material cost.

**How to cite this article:**

Talreja N, Singla S, Shashikiran ND. Comparative Evaluation of Bond Strength and Microleakage of Standard and Expired Composite at Resin-Dentin Interface: An *in vitro* Study. Int J Clin Pediatr Dent 2017;10(1):1-4.

## INTRODUCTION

The use of dental resin composites in dentistry is ubiquitous.^1^ It forms a mainstay in the majority of restorative practices nowadays due to its property of esthetics and advantage of adhesive technology. The property of dental resin composite revolves around each individual component and its concentration. Apart from the components, one of the factors essential for a successful outcome in case of composite resin restorations is the skill of the practitioner and technique of placement. Therefore, considering all the factors it is imperative that dentists understand the rationale for material idiosyncrasies in order to optimize the adhesive interface between the composite restoration and the tooth substrate.

Thus, the development and implementation of various properties of composite dental restorative materials relies on a comprehensive understanding of each component of the material which basically depends on the shelf life of the material, which is the period from the date of manufacturing, for which a material retains the physical and mechanical properties necessary to accomplish its prescribed purpose.^2^ Many a times, in private practice due to delayed delivery and in professional colleges due to lack of supply through proper channels, the practitioners are compelled to use the expired material without any knowledge of the extent of consequences. Sometimes, in spite of all the precautionary measures, the expired material can be used by the clinician inadvertently.

Properties that change over 3 to 4 years may not be necessarily clinically noticeable, but may impact the longevity of the restoration. All these disadvantages increase or are being affected till what extent is still not clear. Despite the growing popularity, all these concerns regarding composites label them as being toxic as it is said to release certain components over a certain period of time.^1^ This is not to say that expired visible light-cured composite is completely worthless. Although not recommended for permanent restorations, other uses for expired composite may be considered. With some finesse, expired composite may be used to fabricate temporary crowns. Expired composite may also be used to repair margins on composite temporary crown materials. Bulk amounts of visible light-cured composite may also be used to stabilize matrix bands for complex amalgams.

Thus, taking into consideration the limited utility of the expired composites the aim of the study was to evaluate and compare the bond strength and microleakage of standard and expired composite at resin-dentin interface.

## MATERIALS AND METHODS

Forty recently extracted noncarious, intact, human permanent maxillary anteriors were selected. Teeth with restoration, cracks, or other structural defects were excluded from the study. They were then divided into two groups (n = 20) for the evaluation of shear bond strength and microleakage respectively. Further, they were subgrouped (n = 5) into four types depending on the composite and bonding agent used:

- *Subgroup I:* Standard composite and bonding agent
- *Subgroup II:* Expired composite and bonding agent
- *Subgroup III:* Standard composite and expired bonding agent
- *Subgroup IV:* Expired composite and standard bonding agent.

### Group I: Preparation and Grouping of the Specimens for Shear Bond Strength

**Figs 1A and B:**
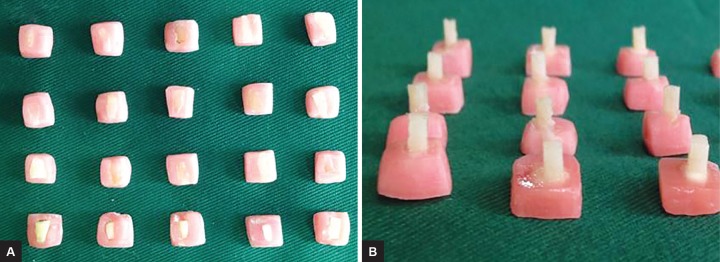
(A) Mounting of specimens for shear bond strength; and (B) Composite resin build-up

- *Mounting of specimens:* Selected specimens were decoronated and sectioned mesiodistally. They were then mounted in acrylic resin in a mold of dimensions 8 × 9 × 10 mm. The labial surface of teeth were ground with a mechanical grinder to obtain a flat dentin surface and were subsequently polished for 30 seconds with wet 240-, 400-, and 600-grit silicon carbide abrasive paper (Fig. 1A).
- *Composite resin build-up*: After the application bonding agent (3M ESPE) on the mounted specimens, composite Filtek Z350 (3M) was placed in increments, using a Teflon mold measuring 2 × 2 mm and cured for 20 seconds on all the 20 specimens (Fig. 1B).
- After placing the prepared samples in distil water bath at 37°C for 24 hours, the specimens were thermocycled for 500 cycles between water baths held at 5°C and 55°C with a 30-second dwell time in each bath and a transfer time of 2 seconds.
- Shear bond strength was measured with an Instron Universal Testing Machine (Model 4444, Instron Corporation, Canton, MA, USA) using the Series IX Software System (Instron Corp) to record the data. A knife-edge shearing rod with a crosshead speed of 5 mm/minute was used to load the specimens until fracture.

### Group II: Preparation and Grouping of the Specimens for Microleakage

**Fig. 2:**
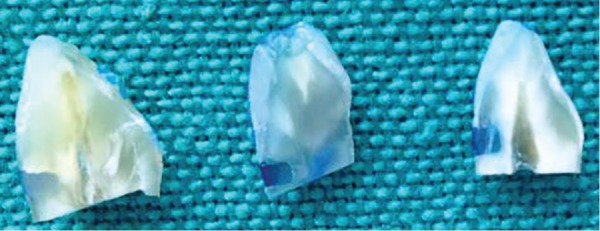
Buccolingual section of the specimens

- *Sample Preparation:* Class V cavity was prepared on labial surfaces of all teeth with a No. 12 diamond round fissure bur under water spray. The gingival margin of the cavity extended into cementum 1 mm below the cementoenamel junction. The cavity dimensions were 3 mm in width (mesiodistal), 2 mm in height (occlusogingival), and 2 mm in depth. The cavities were cleaned, using pumice paste, and then were rinsed with a water spray and gently dried. Dentin was etched with Super Etch gel (37% phosphoric acid) for 15 seconds, and etching gel was applied to all of the prepared cavity wall approximately 0.5 mm beyond unprepared tooth surface using dispensing tips for application. The etching gel then removed with water spray for 10 seconds. Bonding agent was applied according to manufacturer’s instructions and composite placed in two increments and light-cured for 20 seconds.
- Oral conditions were simulated as mentioned above. The teeth were dried and sealed with nail varnish, 1 mm short of the margins of each restoration. The coated teeth were then immersed into a 2% solution of methylene blue for 24 hours.^3^
- Following this, all the samples were sectioned bucco-lingually through the center of the restorations with a slow-speed water-cooled D+Z diamond disk (Fig. 2). Finally, they were visually examined for dye penetration along cavity walls by using a stereomicroscope (Olympus at a magnification of × 40).

All data were processed using Statistical Package for the Social Sciences (SPSS) 10.0 software package (SPSS Inc., Chicago IL, USA) and were analyzed statistically using Kruskal-Wallis test and Mann-Whitney U test.

## RESULTS

**Figs 3A to D:**
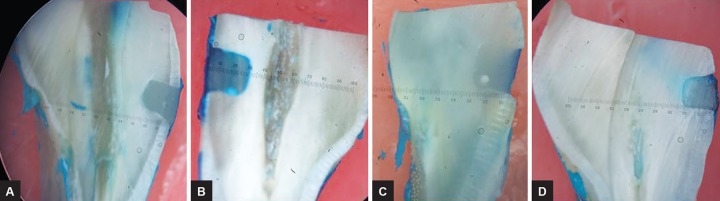
(A) Specimen of group I (mean score of microleakage 0); (B) specimen of group II (mean score of microleakage 3); (C) specimen of group III (mean score of microleakage 3); and (D) specimen of group II (mean score of microleakage 2)

Figures 3A to D show the microleakage exhibited by the samples as seen in the stereomicroscope (40X) along with the scoring criteria.^4^ Table 1 shows the mean value ± standard deviation of maximum load, bond strength, and microleakage scores for all the groups with minimum bond strength for group III and maximum microleakage for group II. Kruskal-Wallis test indicates a significant H-value (maximum load: 7.952; bond strength: 8.298; microleakage: 11.604) among them. For intergroup comparison, Mann-Whitney U test was applied (Table 2) which shows significant values only for two comparisons (group I *vs* group III; group I *vs* group IV) for maximum load and bond strength. For microleakage, it was significant for group I *vs* group II also.

**Table 1:** **Table 1:** Mean and standard deviation (SD) of maximum load, bond strength, and microleakage using Kruskal-Wallis test

		*Maximum load*				*Bond strength*				*Microleakage*			
*Groups*		*Mean*		*SD*		*Mean*		*SD*		*Mean*		*SD*	
I		57.74		4.05		8.91		0.86		0		0	
II		35.62		26.55		5.89		4.17		3.00		1.41	
III		29.36		8.50		4.43		1.41		3.00		1	
IV		35.93		3.32		5.77		0.57		2.00		0	
		H-value		7.952*		H-value		8.298*		H-value		13.300*	

*Significant

**Table 2:** **Table 2:** Intergroup comparison of groups for maximum load, bond strength and microleakage scores using Mann-Whitney U test

		*Maximum* *load*		*Bond* *strength*		*Microleakage*	
Normal composite *vs* expired composite		5.000		5.000		0.000*	
Normal composite *vs* normal composite expired bonding agent		0.000*		0.000*		0.000*	
Normal composite *vs* expired composite normal bonding agent		0.000*		0.000*		0.000*	
Expired composite *vs* normal composite expired bonding agent		7.000		7.000		12.000	
Expired composite *vs* expired composite normal bonding agent		5.000		5.000		7.500	
Normal composite expired bonding agent *vs* expired composite normal bonding agent		7.000		6.000		5.000	

‘Significant, p < 0.001

## DISCUSSION

Over several past decades composites have been widely used on account of esthetic properties. But, the associated concerns with it like microleakage and postoperative sensitivity still prevail and produce dilemma among the clinicians regarding its use in various situations. All these concerns are said to be associated with standard composites. So, it is justified to think that these properties will further deteriorate on using expired composites. But despite of composites being expired, the high cost of this material compels the clinicians to use it without knowing the consequences. Thus, taking into account the high popularity of this extravagant material, the properties of microleakage and shear bond strength of expired composite were evaluated and compared with the standard composite in different combinations with the focus on the extent of use of expired composites.

It has been postulated that minimum bond strength of 17 to 20 MPa is needed to resist contraction forces of resin composite materials, for enamel and dentin.^5^ Clinical experiences confirm that this bond strength is sufficient for successful retention of resin restoration. In the present study, the mean values for bond strength were minimum for subgroup III (standard composite expired bonding agent; 29.39 ± 8.50) and the mean values of microleakage scores were maximum for group II (expired composite and bonding agent; 3 ± 1.41); and group III (3 ± 1). The common factor among these groups is the expired bonding agent, so the values can be attributed to the unfavorable and less predictable bonding between dentin and composite^6^ which was further deteriorated by the expired bonding agent. However, the values of shear bond strength were significant only for two comparisons (Table 2). The lack of significant changes in shear bond strength may be attributed to the short time past after the expiration date^7^ and also to the storage conditions (following manufacturers’ recommendations) of all tested materials until their use. Thus, with the above discussion, it can be anticipated that the property of the formation of a reliable bond between the composite and dentin is not altered on using expired composites and normal bonding agent shortly past the expiration date.

On intergroup comparison, the results for microleakage were statistically significant between the normal and the expired composite whereas they were not significant for shear bond strength. The change in microleakage of expired composite may be explained by incomplete cure of the polymer, perhaps due to degradation, over time, of the components involved in the polymerization of this material.^7^

The mechanical properties of light-cured composites are also highly dependent on the content of the filler particles in the matrix and the extent of polymerization. Some studies^8^ reported decrease in the filler particles of restorative composites kept in liquid medium for certain periods and debonding of the filler particles is due to leakage of its ions into the uncured monomer.^7^ This is affected by the concentration of the photoinitiator^9^ that would suffer degradation throughout the shelf life of the material. For instance, if the silane coupling agent has deteriorated, a composite could suffer from a wear rate greater than what is normally expected.

The number and diversity of processes by which composite resins may be degraded in the oral cavity are huge and are now recognized as a complex interplay of interactions:^1^

- Increasing apprehension arises regarding the safe clinical application of these materials due to their biodegradation under the oral environment.
- Several factors are responsible biodegradation, such as saliva characteristics, chewing, thermal, and chemical dietary changes.

Hondrum^10^ has published research detailing that a visible light-cured composite retains specific physical properties for up to 7 years. Although the utilization of expired dental materials is not recommended, the properties of the material affected by aging are still not clear. Although we recommend an extension in the shelf life of these expensive materials, but how much past the expiration date they can be effectively used should be confirmed by further research, along with evaluating other desirable properties.

## CONCLUSION

Despite the fact that composite shows best properties when it is not expired, in situations when the availability is questionable, the expired composite Filtek Z350 (3M) with normal bonding (3M ESPE) agent can be used in clinical situations.
